# A biopsy-sized 3D skin model with a perifollicular vascular plexus enables studying immune cell trafficking in the skin

**DOI:** 10.1088/1758-5090/ad5d1a

**Published:** 2024-07-12

**Authors:** Krutav Rakesh Shah, Laura Garriga-Cerda, Alberto Pappalardo, Leila Sorrells, Hun Jin Jeong, Chang H Lee, Hasan Erbil Abaci

**Affiliations:** 1 Department of Dermatology, Columbia University Irving Medical Center, New York, NY 10032, United States of America; 2 Department of Biomedical Engineering, Columbia University, New York, NY 10027, United States of America; 3 Regenerative Engineering Laboratory, Columbia University Irving Medical Center, New York, NY 10032, United States of America

**Keywords:** engineered skin, vasculature, hair follicles, skin-on-a-chip, bioprinted skin, immune cells, microphysiological systems

## Abstract

Human skin vasculature features a unique anatomy in close proximity to the skin appendages and acts as a gatekeeper for constitutive lymphocyte trafficking to the skin. Approximating such structural complexity and functionality in 3D skin models is an outstanding tissue engineering challenge. In this study, we leverage the capabilities of the digital-light-processing bioprinting to generate an anatomically-relevant and miniaturized 3D skin-on-a-chip (3D-SoC) model in the size of a 6 mm punch biopsy. The 3D-SoC contains a perfusable vascular network resembling the superficial vascular plexus of the skin and closely surrounding bioengineered hair follicles. The perfusion capabilities of the 3D-SoC enables the circulation of immune cells, and high-resolution imaging of the immune cell-endothelial cell interactions, namely tethering, rolling, and extravasation in real-time. Moreover, the vascular pattern in 3D-SoC captures the physiological range of shear rates found in cutaneous blood vessels and allows for studying the effect of shear rate on T cell trafficking. In 3D-SoC, as expected, *in vitro*-polarized T helper 1 (Th1) cells show a stronger attachment on the vasculature compared to naïve T cells. Both naïve and T cells exhibit higher retention in the low-shear zones in the early stages (<5 min) of T cell attachment. Interestingly, at later stages T cell retention rate becomes independent of the shear rate. The attached Th1 cells further transmigrate from the vessel walls to the extracellular space and migrate toward the bioengineered hair follicles and interfollicular epidermis. When the epidermis is not present, Th1 cell migration toward the epidermis is significantly hindered, underscoring the role of epidermal signals on T cell infiltration. Our data validates the capabilities of 3D-SoC model to study the interactions between immune cells and skin vasculature in the context of epidermal signals. The biopsy-sized 3D-SoC model in this study represents a new level of anatomical and cellular complexity, and brings us a step closer to generating a truly functional human skin with its tissue-specific vasculature and appendages in the presence of circulating immune cells.

## 1. Introduction

Human skin vasculature has a distinct anatomy with two interconnected horizontal vascular plexuses, namely the deep plexus and the superficial plexus, from which capillary loops extend between the epidermal ridges and into the dermal papilla of the hair follicles. The skin vasculature has unique functions, such as controlling constitutive lymphocyte trafficking at homeostasis unlike other non-lymphoid organs [1, 2]. Therefore, perturbations in skin vasculature significantly affect immune cell recruitment [1] and stem cell maintenance [3], contributing to the progression of skin diseases, including psoriasis and atopic dermatitis [4], and to cutaneous infections and wound healing.

Leukocyte homing in skin has classically been studied using real-time intravital multiphoton microscopy on anesthetized rodents [5]. The use of animal models creates many practical limitations on experimental design and may not translate to human T-cell and endothelial cell (EC) interactions. In particular, in animal models it is challenging to monitor the specific immune cell infiltration events, namely tethering, rolling, and extravasation. Moreover, it is impractical to uncouple the cell-cell interactions *in vivo* such as direct modulatory effects of epidermal signals on ECs independent of their effects on T cells. Therefore, it is important to have an *in vitro* human skin model that mimics the circulation of T cells through skin vasculature in 3D and allows for real-time observation of human T-cell and EC interactions in the skin microenvironment.

The human epidermis is composed of the interfollicular epidermis (IFE) and the epidermal appendages, such as hair follicles, which are closely surrounded by the superficial vascular plexus. There are specific epidermal chemokines secreted by the IFE and hair follicles to differentially mediate immune cell trafficking through skin vasculature. There is currently no model that contains an anatomically-relevant vascular plexus in close proximity to the IFE and hair follicles to study their interactions with the immune cells. Our group and others previously incorporated engineered hair follicles [6–8]; a perfusable vasculature [9, 10]; and immune cells [11, 12] as individual components in 3D skin models. Yet, it remains a technical challenge to include these components simultaneously towards achieving a physiological level of cellular and structural complexity.

In this study, we used digital-light-processing (DLP)-based 3D-bioprinting to generate a miniaturized (6 mm biopsy-sized) 3D skin-on-a-chip (3D-SoC) model that mimics the superficial vascular plexus surrounding engineered hair follicles. The perfusion capabilities of the model further allowed for the real-time and high-resolution monitoring and quantification of T cell tethering, rolling, and extravasation events. The biopsy-sized 3D-SoC model developed in this study brings us a step closer to generating a truly functional human skin with its tissue-specific vasculature and appendages, and provides the means to study the immune cell trafficking to the skin in healthy and inflammatory conditions in the context of human cells.

## 2. Methods

### 2.1. Cell culture

Human keratinocytes (KCs) were isolated from neonatal foreskin and cultured up to passage 3 in KC medium (EpiLife; Gibco #MEPI500CA supplemented with S7, Gibco #S0175 and Antibiotic-Antimycotic Gibco #15240112). Human dermal blood endothelial cells (HDBECs) (PromoCell #C-12211) and GFP-tagged HDBECs (Angio-Proteomie #cAP-0005GFP-PM) were cultured up to passage 3 in Microvascular EC Growth Medium (PromoCell #C-22020). Human dermal papilla cells (DPCs) were isolated from discarded scalp tissues from hair restoration surgery (provided by Dr Robert Bernstein) and cultured in DPC medium (DMEM, Gibco #10566016 with 10% fetal bovine serum, Gibco #16000069). Human naïve and Th1 cells were cultured in T cell medium (RPMI media with GlutaMax and HEPES, Gibco #72400120 supplemented with 10% heat inactivated FBS, Gibco #16000069, 1% 100X MEM amino acids Gibco # 11130051, 1% MEM non-essential amino acids Gibco #11140050, 1% Antibiotic-Antimycotic Gibco #15240112). All cells were kept in a humidified incubator at 37 °C and 5% CO_2_ and the culture medium was replaced every other day.

### 2.2. Polarization of T cells

First, peripheral blood mononuclear cells were isolated from whole blood by density gradient using Ficoll-Paque Premium (Cytiva, #175442020), then naïve CD4+ T lymphocytes were isolated through magnetic-activated cell separation using magnetic microbeads (Miltenyi Biotech #130-094-131) according to the manufacturer instructions. The naive CD4+ lymphocytes were plated in 12-well plates (250 × 10^3^ cells ml^−1^) with anti-CD3/anti-CD28 Dynabeads (Invitrogen #11131D) at a ratio of one bead per cell. The culture medium was supplemented with IL-12 [20 ng ml^−1^] (Peprotech #200-12), and anti–IL-4 neutralizing antibody [5 *μ*g ml^−1^] (Miltenyi #130-095-753) to induce T helper 1 (Th1) polarization. From day 3 of polarization, the culture medium was further supplemented with IL-2 [50 U ml^−1^] (Peprotech #200-2). From day 3 to day 7, 50% of the medium was replaced every day with complete medium, the cell clumps were separated by pipetting, and the cells were split when a density of 2 × 10^6^ cells well^−1^ was reached (usually twice). From day 7 the polarized Th1 cells were maintained in T cell medium supplemented with IL-15 [10 ng ml^−1^].

### 2.3. Flow cytometry

Th1 cells were plated in two wells of a 12-well plate. One well was further activated using the Cell Stimulation Cocktail (eBioscience #00-4975-93) at a 500X dilution and a density of 2 million cells ml^−1^ for 6 h, while the other well was kept in its basal activation state. First, the cells were stained with Live/Dead Violet (Invitrogen # L34963) to exclude the dead cells following the manufacturer’s instructions, then blocked with Human TruStain FcX (BioLegend #422302). The BD Cytofix/Cytoperm fixation and permeabilization Kit (BD Biosciences #554714) was used for the intracellular staining. Prior to fixation, the cell surface markers were stained using Cell Staining Buffer (Biolegend # 420201) with the following antibodies: anti-CD3 V500 (BD Horizon #561416), anti-CD4 Bv605 (BioLegend #300556), and anti-CD8 BUV496 (BD Horizon #612942). Then, the cells were fixed, permeabilized, and stained with the following antibodies for intracellular markers: anti-IFNg APC-eFluor 780 (eBioscience #43-7319-42) and anti-TNFa PE-Vio 770 (Miltenyi Biotec #130-127-531). The compensation was performed using UltraComp eBeads (eBioscience #01222242). All cells were analyzed on Novocyte Penteon 5 Lasers Cytometer (Agilent Technologies) using the software NovoExpress (Agilent Technologies).

### 2.4. Fabrication of the SoC template and perfusion chamber

The CAD model of the SoC template was designed with horizontally aligned microchannels for the vasculature and vertically aligned channels for the hair follicles using the SolidWorks software. The SoC template was 3D-printed using the Lumen-X bioprinter (CELLINK) using GelMA (CELLINK; printing parameters recommended by the manufacturer for 100 *μ*m resolution). The SoC template was then washed for 2–3 d in phosphate-buffered saline (PBS) with antibiotics on a shaker platform, dipped in 75% ethanol for 5 mins, and stored in PBS at 4 °C. The perfusion chamber was designed on SolidWorks software to include an inner chamber to accommodate the SoC (after slight post-swelling in PBS), an inlet and outlet port that connects to the inlet/outlet of SoC through syringe needles with a flexible tip (Optimum; 18 G). The chamber was 3D-printed in polylactic acid (PLA) using a fused deposition modeling 3D-printer (3DWOX1; Sindoh), and attached to a glass slide using a surgical glue (MasterSil912MED; Masterbond). The SoC was placed in the inner chamber and two flexible syringe tips were inserted into the inlet/outlet ports. To completely seal the system, a 4% (w/w/ in PBS) agarose solution was added to fill the gap between the SoC and the walls of the chamber.

### 2.5. Development of the SoC model

The SoC template was submerged in DPC culture medium overnight at 37 °C prior to seeding on the top surface 50 000 DPCs in 30 *µ*l of culture medium per construct. The construct was tilted laterally on all sides for 5 min to facilitate uniform DPC settling in the hair follicle channels and placed in the incubator overnight submerged in DPC medium. Next day, the aggregate formation was confirmed by light microscope and 1 million KCs resuspended in 30 *µ*l of KC culture medium were seeded on the top surface of each SoC. After 5 d of submerged culture, the ECs were seeded into the vascular channels by perfusing 1 × 10^5^ cells resuspended in 100 *µ*l of culture medium. To enhance the ECs attachment on the vascular walls, the SoC was incubated without flow for 15 min, then flipped upside down for additional 15 min to guarantee uniform coverage. Then, the channels were continuously perfused using a peristaltic pump with EC culture medium overnight at 0.5 ml min^−1^ which was increased to 2.4 ml min^−1^ the next morning and kept for 2 d. At that time, the SoC was switched to air-liquid-interface (ALI) culture (perfusion with cornification medium supplemented with 10 ng ml^−1^ VEGF) and maintained for 5–7 d before further analysis. T cells were labeled with CellTracker Far Red (Thermo Fisher) and perfused at a density of 1 × 10^6^ cells ml^−1^. For our experiments including dermal fibroblasts (FBs), we flipped the SoC and seeded 50 000 FBs in 30 *µ*l of medium on the surface, allowed FBs to attach, and submerged in co-culture medium. We allowed FBs to migrate for 4 d before fixing the SoC in 4% paraformaldehyde and proceeding with immunostaining.

To develop the conventional skin constructs, we followed the protocol from our previous studies [13, 14]. Briefly, we first poured 0.2 ml of neutralized and salt-balanced collagen solution made with 3 mg ml^−1^ collagen type I (EMD Millipore, #08-115) in a transwell insert inside a 12 well plate, followed by addition of 165 × 10^3^ FBs resuspended in 0.6 ml of the collagen solution. We submerged the construct in FB medium and allowed remodeling for 1 week before seeding 100 × 10^3^ KCs and submerging in co-culture epidermalization medium [14]. After 5 d, the CSC was transitioned to ALI culture and maintained for 5–7 d.

### 2.6. Nanoindentation measurements

The effective indentation moduli (*E*_Eff_) of GelMA devices with or without cellular components were measured using a PUIMA™ nanoindenter (Optics11, Netherlands) as a modified protocol from our previous work [15]. CSC samples were measured as a control. Briefly, the indentation was carried out employing a probe radius of 10.5 *µ*m and a stiffness of 42.2 N m^−1^. At each indentation location, a maximum force of 10 mN was applied at 10 *µ*m s^−1^, and more than 15 points were randomly selected at different locations on constructs. The indentation locations were controlled by a high-resolution mobile X-Y stage integrated into the system.

### 2.7. Immunofluorescent and confocal imaging

The devices were fixed overnight in 4% paraformaldehyde diluted in PBS at 4 °C and then transferred to 30% sucrose at 4 °C. After 24 h, the devices were embedded in optimal cutting temperature compound and cryosectioned at a thickness of 16 *µ*m. The sections were subsequently stained with Hematoxylin and Eosin (H&E) or processed for immunofluorescent staining.

For immunofluorescence, the sections were dried overnight at room temperature, permeabilized with 0.1% Triton X for 15 min, blocked with 5% donkey serum and 8% bovine serum albumin in PBS for 1 h at room temperature, and incubated with primary antibodies overnight at 4 °C. The following antibodies were used: keratin 14 (BioLegend #906004), keratin 10 (#BioLegend #905404), Ki67 (Abcam #ab16667), loricrin (Abcam #ab198994), involucrin (Abcam #ab27495), desmoglein 1 (Progen #651111), and trichohyalin (Santa Cruz #sc-80607). The sections were incubated at room temperature with the following secondary antibodies: Alexa Fluor 647 (Invitrogen #A78952), Alexa Fluor Plus 594 (Invitrogen #A32754), and Alexa Fluor Plus 488 (Invitrogen #A32766). The nuclei were stained with 4′,6-diamidino-2-phenylindole (BioLegend #422801). Whole mount-staining was performed following the same protocol as above, except 0.1% Triton X was used in all the staining and washing solutions. The primary antibodies used were: keratin 14 (BioLegend #906004), keratin 10 (#BioLegend #905404), loricrin (Abcam #ab198994), versican (Invitrogen #PA1-1748A), LEF1 (Cell Signaling Technology #2230S), Cytokeratin K40/AE13 (Abcam #ab16113), VE cadherin (Abcam #ab232880), CD31 (Abcam #ab28364), Keratin 71 (Invitrogen #PA5-28558), and Phalloidin (Invitrogen #A12381). Ce3D Tissue Clearing solution (BioLegend #427704) was used to clear the samples. All images were acquired using a Leica Stellaris 5 (Leica, Germany).

### 2.8. Endothelial permeability assay

The devices were perfused with a Rhodamine-20 kDa dextran (8 mg ml^−1^) and Fluorescein-40 kDa dextran (80 mg ml^−1^) solution and imaged every 5 min using Leica Stellaris 5 microscope. Control experiments were performed using acellular SoC. The data was analyzed with ImageJ to detect the fluorescence intensity at the central polygon of the vascular pattern. To estimate the endothelial permeability, we performed implicit time-dependent simulations in the ‘diffusion module’ using finite element methods with the ‘normal’ mesh size using COMSOL Multiphysics software v6.1. We changed the permeability values to give the best fit to our experimental data through the least sum of squares method. In order to estimate the diffusivity of dextran molecules in GelMA, we used the data from SoC with acellular microchannels. Next, we used a simplified analytical solution of the Fick’s second law of diffusion, and iterated the diffusion coefficient to give the best fit to the time-dependent concentrations measured for three different distances from the vessel boundary.

### 2.9. Quantification of T cell attachment and infiltration

Images were acquired using the Leica Stellaris 5 microscope, and subsequent analysis was performed using ImageJ software. The following methods were employed to quantify T cell attachment and infiltration dynamics. Initially, all images were converted to 8-bit to standardize pixel intensity. Threshold values were optimized based on predefined parameters to enhance T cell visibility. Images were then converted to binary using the established threshold and all the binary images were processed to generate masks representing T cell regions. For the T cell Retention Analysis, the entire device region was screened for T cells using the circle tool, and this process was repeated for different time intervals. For the Region-Based Retention Analysis individual stress-specific regions within each device were screened using the Free Hand Selection tool. Region-based analysis was conducted for distinct time intervals, providing insights into T cell retention dynamics. Lastly, for T Cell Infiltration Analysis each chamber was simultaneously screened and the infiltrated T cells were analyzed for their distance from the nearest vessel and the total number of infiltrated cells in each device was quantified.

### 2.10. Statistics

The statistical analysis was performed using a two-tailed paired *t*-test with 95% confidence interval with the software GraphPad Prism. For all experiments three SoCs per condition were used. *p* < 0.05 was considered significantly different. Data were shown as means ± SEM. The statistical analysis for the nanoindentation measurements was performed using one-way ANOVA test using GraphPad Prism. Data were represented as a violin plot.

## 3. Results

### 3.1. Biofabricating the 3D-SoC model with a perfusable vascular plexus surrounding hair follicles

**Figure 1.**
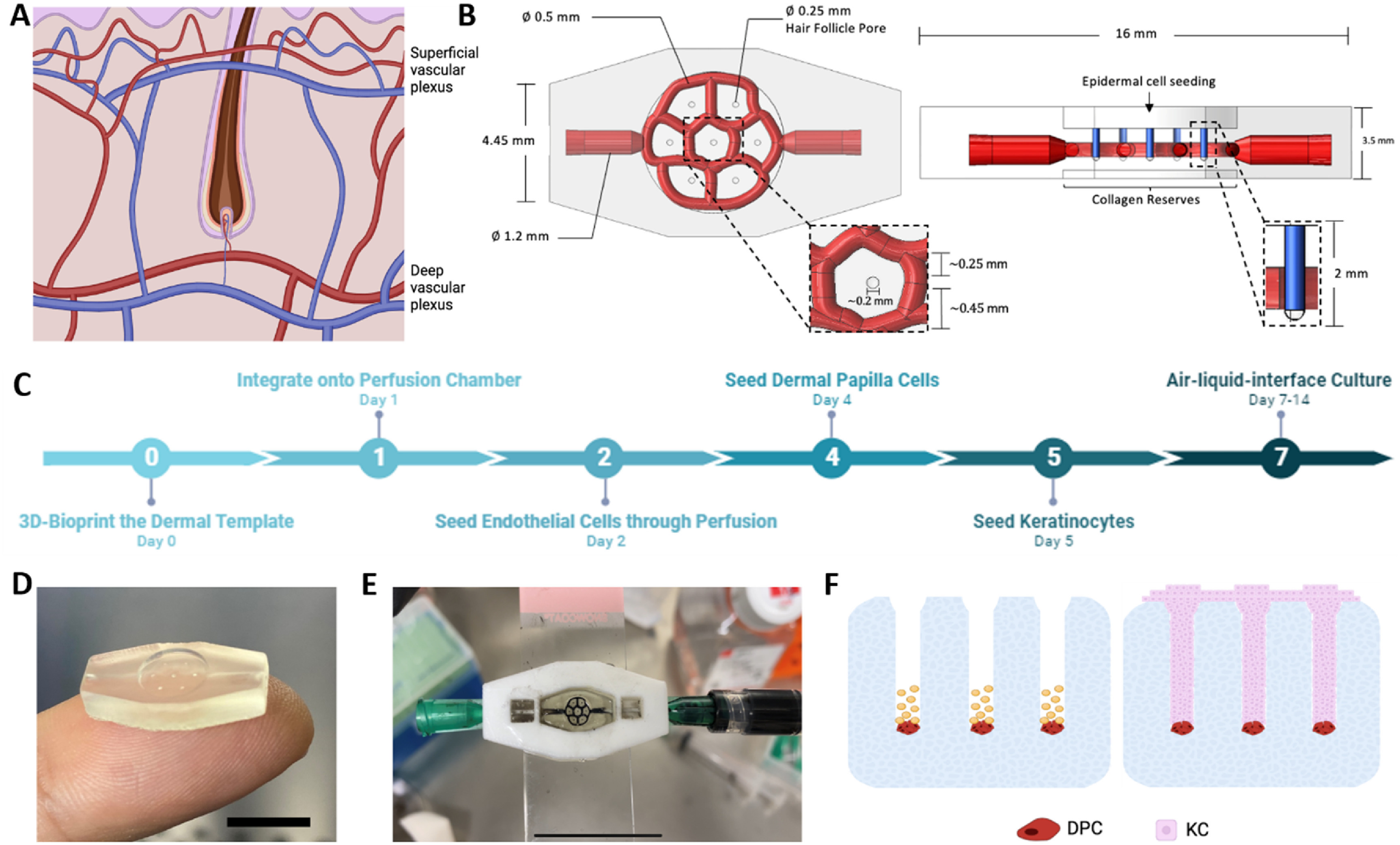
Description of 3D-SoC biofabrication and model development. (A) Two vascular plexuses that run parallel to the skin’s surface make up the human cutaneous vasculature: the superficial plexus, which is located just under the papillary dermis and surrounds the hair follicles; and the deep plexus, which is located in the lower reticular dermis. (B) Top and side views of CAD drawings of the 3D-SoC model developed in SolidWorks depicting the design and dimensions: a 0.5 mm-diameter horizontally-aligned vascular network (shown in red) surrounding the HF; seven vertically-aligned hair follicle microchannels (shown in blue) with a diameter of 250 *µ*m and a depth of 2 mm, matching the physiological range. (C) Timeline of SoC development showing the significant steps of the protocol. (D) Picture of the 6 mm biopsy sized 3D SoC template biofabricated using the CELLINK Lumen X bioprinter with a photocrosslinkable ink composed of GelMA. Scale bar: 1 cm; (E) picture of the SoC integrated into a 3D-printed perfusion chamber attached on a transparent glass slide; to demonstrate the vascular perfusion, black India ink was perfused through the inlet/outlet ports using a syringe. Scale bar: 2 cm; (F) schematic describing the seeding strategy of DPCs and KCs into the hair follicle microchannels. First, a single cell suspension of DPCs was seeded (shown in red) into the HF microchannels. The cells were given time to settle before the remaining area of the pore was filled with KCs (shown in pink) to achieve a physiologically-relevant conformation found in hair follicle development.

Our goal was to design a 3D-SoC system that: (i) had a network of microvessels that is horizontally aligned around each engineered hair follicle, resembling the anatomy of the superficial vascular plexus (figure 1(A)); (ii) included perfusion capabilities to capture a physiological range of shear stresses on dermal ECs; (iii) allowed for real-time and high-resolution imaging of immune cell recruitment and infiltration events; (iv) mimicked both hair follicles and IFE; and (v) was as small as a 6 mm skin biopsy. Based on these criteria, we created a CAD model of the 3D-SoC with the dimensions shown in figure 1(B).

We started by 3D-bioprinting the CAD model (figure 1(C)), as an acellular 3D-SoC template, using a gelatin methacrylolyl (GelMA)-based bioink and a DLP 3D-bioprinter (Lumen-X) (figure 1(D)). To accommodate 3D-SoC and perfuse the vasculature, we 3D-printed a perfusion chamber made of PLA (supp. figure 1), adapted from a previous work [16], and connected the inlet/outlet ports to a peristaltic pump (figure 1(E)). After the integration of 3D-SoC onto the perfusion chamber, the protocol continued by seeding HDBECs through perfusion to cover the inner walls of the vascular channels. After keeping the system under continuous perfusion for 48 h, we seeded human primary DPCs into the vertical hair follicle microchannels where the cells settle down to the bottom and spontaneously form aggregates overnight (strategy depicted in figure 1(F)). The next day, we seeded human primary KCs on the top surface and let them fill up the vertical hair follicle channels and engulf DPC aggregates to achieve a physiologically-relevant conformation of cells in the hair follicle. KCs also covered the area between follicles to form the IFE. After 2–3 d of submerged culture, we brought the 3D-SoC into ALI culture by removing the medium from the top surface and feeding the model only through perfusion to allow for cornification of the IFE.

To ensure the mechanical properties, e.g. stiffness, are in the range of conventional 3D skin models that are typically composed of collagen type I containing dermal FBs and KCs, we measured the effective Young’s modulus using nanoindentation, and showed that GelMA bioink from Cellink without cells has a stiffness of 20.4 kPa (estimated GelMA concentration is between 5% to 10% based on previous literature). When we included the cells and developed the 3D-SoC, the stiffness decreased to 13.2 kPa, suggesting cells remodel the GelMA, getting the stiffness closer to the range of collagen I-based 3D skin models (supp. figure 2).

### 3.2. Establishing the vascular network in 3D-SoC

**Figure 2.**
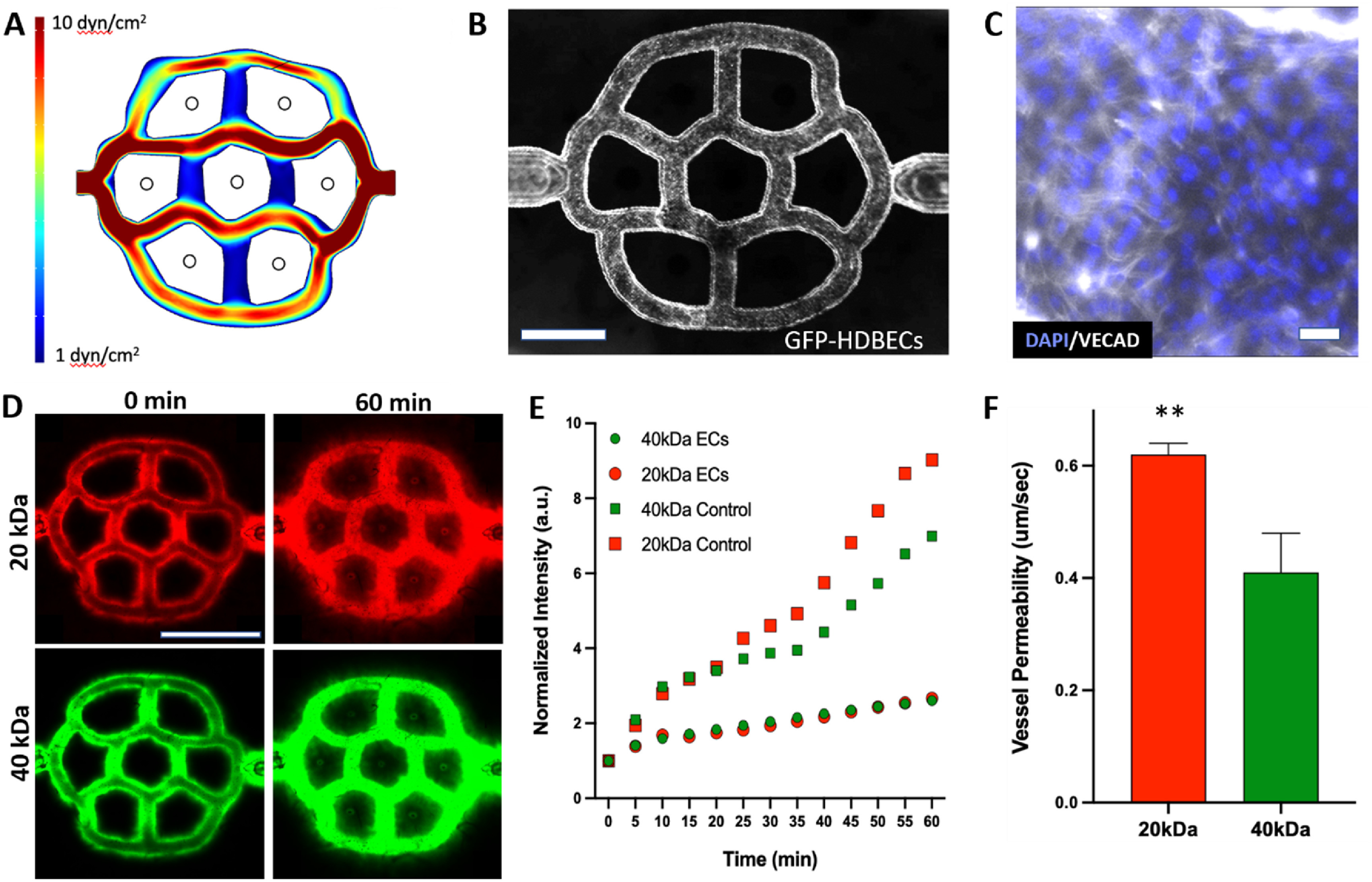
Characterization of the vascular plexus in 3D-SoC. (A) The COMSOL model displays the calculated range of shear stresses in the vascular pattern. The design recapitulates the physiological range of shear stress levels found in the cutaneous capillaries, venules, and arterioles. We divide the vasculature into three shear rate zones as low-shear (vertical interconnecting channels), mid-shear (two outermost channels, top and bottom) and high-shear (two innermost, horizontal channels). (B) Imaging of the vascular network seeded with GFP-HDBECs confirms uniform coverage of the microchannel walls. Scale bar: 1 mm; (C) Immunofluorescent staining of primary HDBECs in 3D-SoC with VE-cadherin (VECAD; white). Scale bar: 5 *µ*m; (D) confocal imaging of the 3D-SoC seeded with HDBECs perfused with both 20 kDa and 40 kDa dextran at time zero and sixty minutes allowing for comparison of the permeability characteristics. Scale bar: 2 mm; (E) the graph shows increased leakage of dextran in the model without HDBECs (acellular control) for both molecular weights. (F) Time-lapse transport data integrated into a COMSOL model enabled the estimation of the average permeability of the vasculature. The permeability values were determined to be 0.62 *µ*m s^−1^ for 20 kDa and 0.41 *µ*m s^−1^ for 40 kDa respectively (** = *p* < 0.01).

We performed COMSOL simulations to calculate the levels of shear stress throughout the vascular network in 3D-SoC, and found that we can achieve shear stress levels from 1 to 10 dyne cm^−2^, mimicking the physiological range found in human skin arteries, arterioles and venules [17] (figure 2(A)). Following this analysis, we identified three shear zones: a high shear, a medium shear and a low shear. This is an important feature to simultaneously study the effect of shear and flow rate on biological processes, including immune cell infiltration. Most immune cell trafficking in the body occurs through the post-capillary venules (low shear region), where the effect of shear on this process is poorly understood. Here, we endothelialized the inner walls of the vascular network with GFP-HDBECs and confirmed that they cover the entire surface area uniformly throughout the network (figure 2(B)), and establish adherent junctions (figure 2(C)).

To confirm their endothelial barrier function, we perfused the endothelialized network with 20 kDa Rhodamine-tagged Dextran and 40 kDa fluorescein-tagged dextran molecules simultaneously, and measured their diffusion from the lumen to the avascular skin region over time using confocal microscopy (figure 2(D)). We found that endothelialized vessels significantly delay the transport of molecules, compared to acellular channels, in a size-dependent manner (figure 2(E)). Integrating the time-lapse transport data into a COMSOL model, we estimated the average permeability of the vasculature to be 0.62 *µ*m s^−1^ and 0.41 *µ*m s^−1^ for 20 kDa and 40 kDa, respectively, (figure 2(F)), mimicking the selective permeability of blood vessels [18, 19]. In addition, we estimated the diffusion of 20 kDa and 40 kDa in the dermis to be 16 *µ*m^2^ s^−1^ and 53 *µ*m^2^ s^−1^, respectively.

### 3.3. Validating the follicular and interfollicular epidermal components

**Figure 3.**
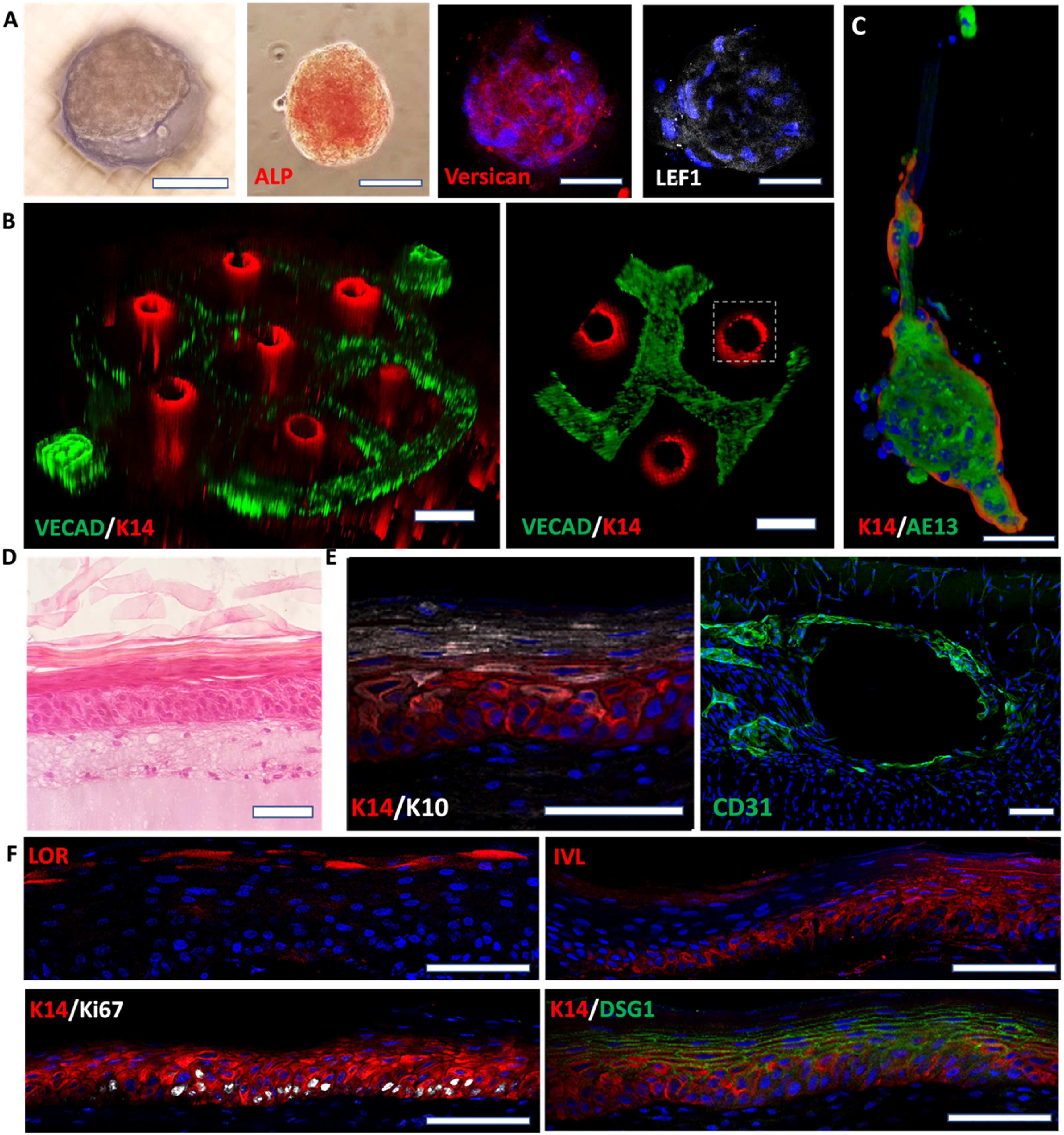
Characterization of the follicular and interfollicular epidermis. (A) Immunofluorescence staining of DPC aggregates at the bottom of the vertical hair follicle microchannels in the SoC model; the expression of alkaline phosphatase (ALP), versican, and LEF1 is associated with functional hair-inductive DPCs. (B) The anticipated spatial distribution of cell types in the printed vascular plexus and hair follicle channels is demonstrated by staining for endothelial cell marker, VECAD (green), and basal epidermal/hair follicle marker, Keratin 14 (K14; red). (C) The proper conformation of the follicular component is further demonstrated by the expression of the outer and inner root sheath markers, K14 and AE13, respectively. (D) H&E of the 3D-SoC showing the interfollicular epidermal compartment and the full development of all epidermal layers. (E) Representative immunofluorescence staining of whole-mount tissues (orthogonal views) showing K10 and K14 expression patterns (left), and CD31 staining to display endothelial cell coverage of the perfusable vasculature (right). (F) Tissue sections showing proper epidermal maturation with basal expression of K14 and Ki67, suprabasal involucrin (IVL) and desmoglein 1 (DSG1), and loricrin enhancing the terminally differentiated layer. The presence of Ki67^+^ proliferative keratinocytes indicate the homeostatic state of the epidermis and its self-renewal capability. Scale bars: 100 *µ*m for (A), (C), (D)–(F); 250 *µ*m for (B).

Using the strategy depicted in figure 1(D), we seeded DPCs followed by KCs in the 3D-SoC. The DPCs formed spontaneous aggregates and restored the expression of markers, such as Versican, and exhibited alkaline phosphatase activity (figure 3(A) and supp. figure 3) which are associated with their hair inductive properties [20]. Next, we showed that both the vascular and follicular epidermal components are maintained in 3D-SoC with the vasculature surrounding the K14+ basal layer of the follicular epidermis (figures 3(B) and (C)).

Immunostaining for K14, a marker for the outer sheath of HFs, and AE13, a marker for the inner root sheath, showed that KCs can differentiate into specific hair follicle lineages with a physiologically-relevant organization of the layers (figure 3(C)). Additional staining with Keratin 71 confirms the inner root sheath differentiation of the engineered HFs (supp. figure 4). To characterize the IFE, we performed H&E staining (figure 3(D)) and immunostaining for Keratin 14 (basal layer), Keratin 10, Involucrin and Desmoglein 1 (spinous layer), and Loricrin (granular layer) (figures 3(E) and (F)), indicating the proper terminal differentiation and cornification of the IFE. Overall, this data demonstrated that 3D-SoC can model the superficial vascular plexus with perfusion capabilities in close proximity to the follicular epidermis and IFE. To further test whether FBs can be added into the model in the future, we seeded them on the bottom surface of our 3D-SoC and allowed them to proliferate for 4 d. We observed FB migration from the surface into the dermal compartment, demonstrating that FBs can be successfully incorporated into our model (supp. figure 5).

### 3.4. Incorporating and monitoring the infiltration of circulating immune cells to 3D skin

**Figure 4.**
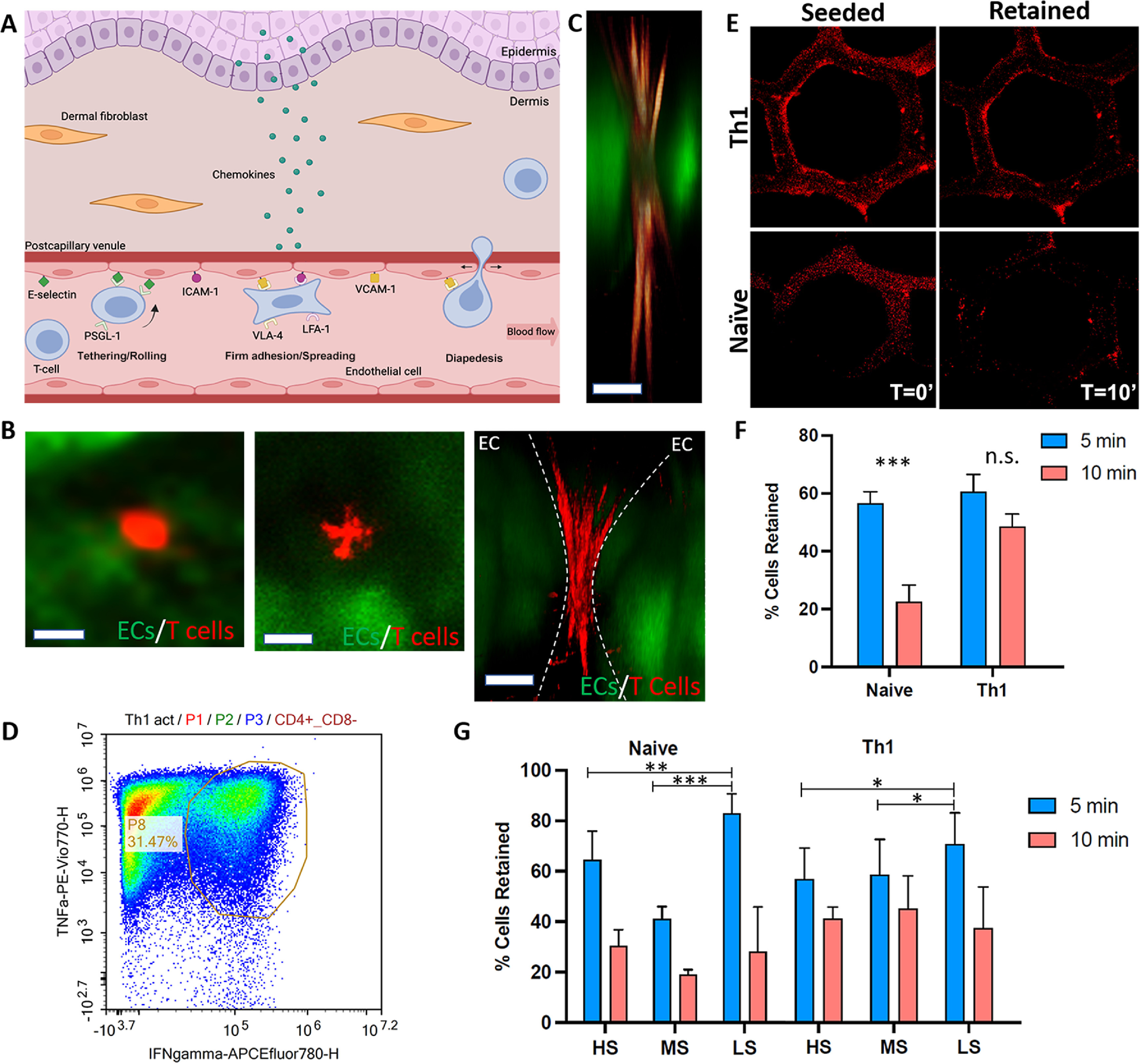
Incorporation and real-time monitoring of circulating T cells in 3D-SoC. (A) Schematic representation of the stages of T cell infiltration into human skin. (B) Live immunofluorescent images showing the naïve T cells labelled with CellTracker (red) on HDBECs in the first 1–2 min (left panel; the round morphology resembles the tethering/rolling stage); between 2–5 min (middle panel; the spread morphology resembles the firm adhesion stage); and between 5–15 min (right panel; the morphology and location relative to ECs resembles the diapedesis stage). The first two images show the top view, and the right-most image shows a cross-section of the 3D-SoC. Scale bars: 5 *µ*m; (C) High magnification image capturing a T cell (red) with its lamellipodia squeezing between two endothelial cells (green), resembling the morphology of T cells *in vivo* during their movement through capillary walls (namely diapedesis). Scale bar: 2 *µ*m; (D) characterization of Th1 cells polarized from Naïve T cells *in vitro* through flow cytometry showing expression of both Interferon γ and TNFα. (E) Comparison of the attachment of the T cells to the shear stress analysis for naive and Th1 cell population. (F) Total percentage of naïve T cells and Th1 cells retained after 5 and 10 mins of flow. (G) Percentage of cells retained for distinct shear zones; HS: high-shear, MS: mid-shear, LS: low-shear. (* = *p* < 0.05, ** = *p* < 0.01, *** = *p* < 0.005).

The entry of T-lymphocytes into the skin is finely regulated by a cascade of interactions between the circulating T-lymphocytes and skin vascular ECs (non-lymphatic ECs). During tethering, the first interaction of this process, T-cells make contact with ECs and then they start rolling on their surface, while maintaining their round morphology. This is followed by firm adhesion of T-cells on the EC monolayer and spreading, finally leading to diapedesis (the migration of T cells through gaps between ECs) (figure 4(A)). When we perfused 3D-SoC with naïve T cells labeled with CellTracker, we were able to capture in real-time the T cell morphologies resembling individual T cell infiltration stages (figure 4(B)). During the rolling stage that took place in the first 1–2 min of cell contact, the T cells displayed a round morphology; then over the next 2–5 min they spread on the ECs; and finally, they infiltrated the intercellular gaps between ECs (supp. figure 6), indicative of diapedesis. In this final stage, the T cells exhibited a typical morphology with dynamic protrusions, e.g. filopodia, which we were able to capture in high resolution in 3D-SoC (figure 4(C)).

Our next goal was to examine the adherence of naïve T cells on ECs in comparison to polarized T helper 1 (Th1) cells, which are mature effector lymphocytes involved in inflammatory skin conditions, such as psoriasis. We adapted a previously established protocol to polarize naïve T cells into Th1 cells through IL12 and anti-IL4 treatment [11] and confirmed via flow cytometry that ∼32% of cells expressed both Interferon γ and TNFα, compared to nearly zero expression detected in naïve T cells (figure 4(D) and supp. figure 7). The multicellular environment in the 3D-SoC does not seem to have a negative effect on the Th1 phenotype (supp. figure 8). We perfused the 3D-SoC either with naïve T cells or Th1 cells labeled with CellTracker, and monitored their adherence on the vasculature over 10 min under perfusion (figure 4(E)). Quantification of the images showed that 55% of naïve T cells and 60% of Th1 cells were attached after the first 5 min of perfusion, whereas only 20% of naïve T cells remained attached after 10 min (63% decrease), a significantly lower number compared to Th1 cells for which we observed a 45% after 10 minutes (25% decrease) (figure 4(F)). Finally, we analyzed the data for different shear rate zones in the SoC to see whether there is a correlation between blood flow dynamics and T cell attachment. The data indicated that there is significantly higher retention of both naïve T cells and Th1 cells in the low-shear zone, compared to the high-shear and mid-shear zones in the first 5 min (figure 4(G)), whereas the effect of shear rate on naïve or Th1 cell retention was lost after 10 min of perfusion.

**Figure 5.**
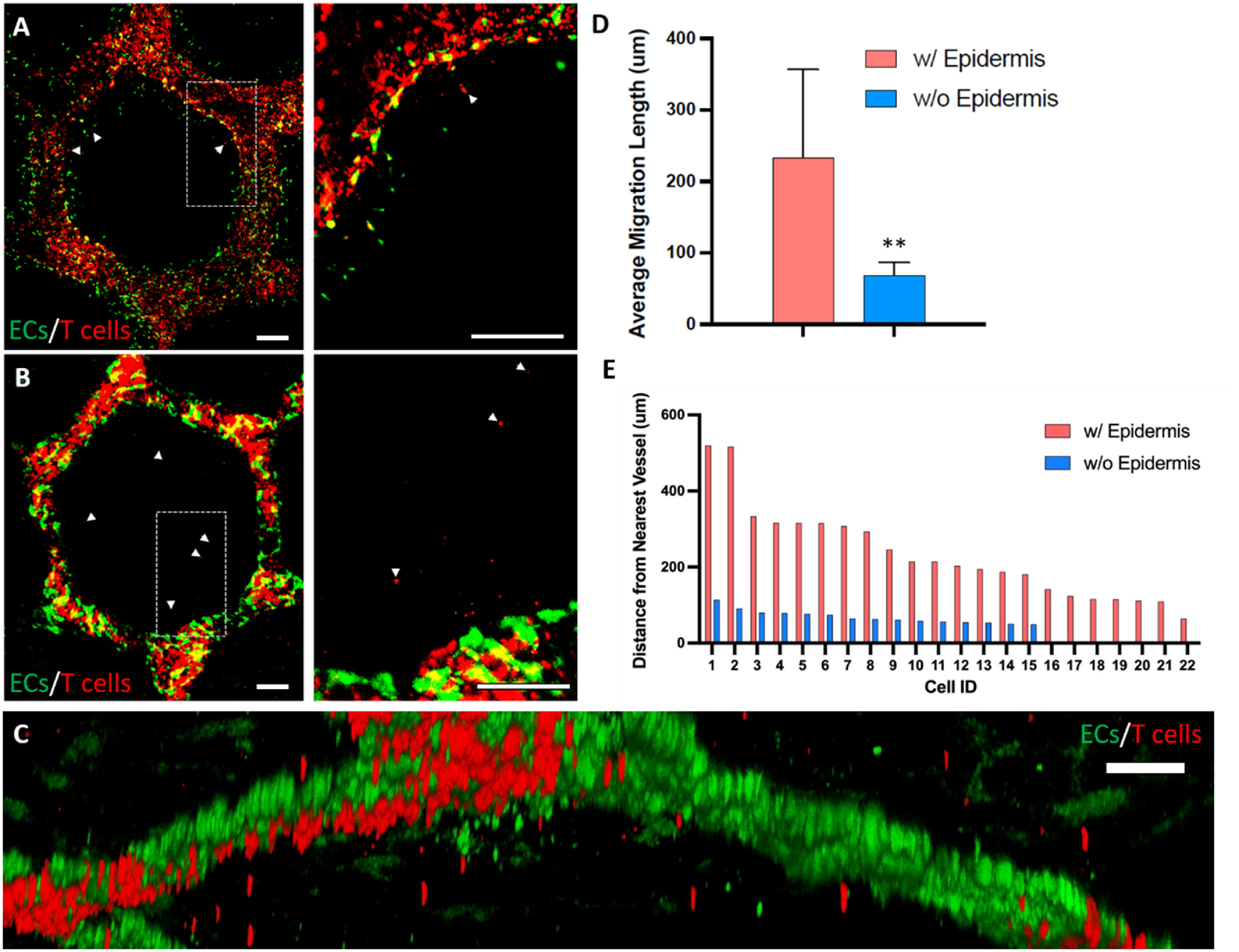
Effect of the epidermis on Th1 cell infiltration into the dermal compartment. The infiltrated Th1 cells (labeled with CellTracker red) 5 d after their introduction into 3D-SoC without epidermis (A) and with epidermis (B). Arrow heads point to infiltrated Th1 cells; right panels show the zoomed in image of the dashed windows shown on the left panels. (C) 3D reconstructed high-magnification image showing the transmigration of Th1 cells in 3D-SoC with the epidermis. (D) Average migration length for T-cells in the presence and absence of epidermis (** = *p* < 0.01). (E) The distance of each infiltrated Th1 cell from the nearest vessel in the presence and absence of epidermis. Scale bars: 250 *µ*m.

In human skin, epidermal chemokines play an important role to facilitate T cell trafficking [21]. To test whether 3D-SoC can mimic the effect of epidermal cues on T cell infiltration into the dermis surrounding the HF structure, we cultured the Th1-perfused SoCs for 5 days and quantified the number of Th1 cells transmigrated into skin in the presence and absence of the epidermis (figures 5(A)–(C) and supp. figure 9). We first quantified the distance of transmigrated Th1 cells from the vascular border, and found an average distance of 233 *µ*m (figure 5(D)) when the epidermis is present and 68 *µ*m when the epidermis is absent. The maximum distance travelled by Th1 cells was also significantly higher in the presence of the epidermis where Th1 cells were detected as far as 520 *µ*m from the nearest vessel (figure 5(E)). When the epidermis was absent, the maximum migration length dropped to 113 *µ*m with a total number of 15 transmigrated cells detected compared to 22 cells when the epidermis was present. Overall, this data highlighted the role of epidermal signals on T cell transmigration and infiltration, and validated the capabilities of 3D-SoC to study the interactions between immune cells and skin vasculature in the context of epidermal signals.

## 4. Discussion

Developing a physiologically-relevant model of skin is a bioengineering challenge considering the cellular and structural complexity of human skin with more than 50 different cell types and appendages. In this study, DLP technology allowed us to advance 3D skin models to a new level of complexity to include five different skin cell types, and anatomically complex structures, such as the vascular plexus and hair follicles, while reducing the size down to a 6 mm- skin punch biopsy. Furthermore, our miniaturized 3D-SoC model allows for circulating immune cells and monitoring immune cell infiltration in high-resolution and real-time, providing an advanced and cost-effective research tool to investigate skin inflammatory responses in the axis of follicular epidermis, IFE, endothelium and immune cells.

In healthy individuals, nearly 20 billion T lymphocytes populate the entire skin surface, twice the number of T lymphocytes found in the blood circulation [22]. In contrast to other non-lymphoid tissues, T cells constantly traffic to and within the skin, patrolling for pathogen invasion and inflammatory conditions [1, 2]. In inflammatory conditions, such as psoriasis, T cells are polarized and activated, e.g. Th1, and infiltrate the skin at higher rates. In our 3D-SoC model, we observed that naïve and Th1 cells initially attached to the ECs at a similar rate, but after 10 min a higher number of Th1 cells retained on the vascular surface, suggesting that firmer bonds formed after the first 5 min of tethering may be more determining for sustained T cell attachment on ECs. The shear rate exerted by the blood flow on T cells is an important factor affecting T cell retention on the endothelium [23]. There is a wide range of shear stress experienced by cells in the cutaneous arterioles, capillaries and venules. The vascular pattern in 3D-SoC captures this wide physiological range and allows for studying the effect of shear rate on T cell attachment. In our study, we detected a higher retention of both naïve and T cells in the low-shear zone within the first 5 min, which at later time-points became independent of the shear rate levels. This data suggests that shear rate may play a more major role in the process of selectin binding during the initial rolling/tethering events, compared to later stages in which T cells firmly adhere to ECs through integrin binding. The majority of T cell infiltration in the skin occurs at post-capillary venules. This higher rate of infiltration is typically attributed to their structural properties and specialized EC phenotypes [24, 25]. Our data suggests that shear rate, which is low in post-capillary venules, can be a significant and overlooked factor determining the location of T cell infiltration in the skin.

Several chemokines, e.g. CXCL16 and CCL18, secreted by the epidermis are known to play a role in promoting T cell infiltration to the skin during homeostasis and inflammation [21]. Our previous studies also highlighted the indispensable role of epidermal signals in T cell migration in the dermal ECM of a 3D skin model, albeit without a vasculature [11]. Here, our data confirmed that the presence of the epidermis increases migration distance of Th1 cells from the vessels towards the epidermis. Although we found an increasing trend in the total number of transmigrated T cells in the presence of the epidermis, it was not a statistically significant difference. This may suggest that epidermal signals contribute to the migration of T cells in the dermal ECM towards the epidermis rather than affecting their transendothelial migration from the circulation.

Our lab and others made significant progress in the past to include individual skin components, such as skin appendages and vasculature [6, 7, 9, 10]. Herein, the ability gained to integrate and control the spatial organization of these two major components of the skin is not only important for skin disease modelling or drug testing, but may also have implications in skin replacement therapy. The close proximity of ECs and hair follicles has been shown to promote hair regeneration [26], and assist in EC survival and proliferation [27]. In our previous work, we pre-vascularized 3D skin constructs containing engineered human hair follicles through spontaneous capillary formation (e.g. no patterning or perfusion), and showed that pre-vascularization of hair-bearing 3D skin grafts is required for the growth of human hair follicles in mice. Preconditioning of the vessels via perfusion prior to engraftment was recently shown to improve infarcted heart healing [28]. Additionally, vascularization of 3D skin constructs can prevent formation of a necrotic core [29]. Therefore, having a perfusable vasculature in close proximity to hair follicles in 3D-SoC may further improve the viability of hair-bearing 3D skin grafts, although awaiting further investigation. Despite the anatomically-relevant organization of the vasculature in 3D-SoC, the diameter of the vasculature is still larger than that of human skin microvessels. This is due to limitations of the 3D-bioprinting system we used, and can be addressed with the emerging bioprinting approaches [30]. In addition, the method developed here to biofabricate hair follicles can potentially be extended for creating rete ridges and other appendages, such as sweat glands.

Overall, this study describes a novel 3D skin model for the first time, to our knowledge, to include an anatomically-relevant vascular plexus integrated with bioengineered skin appendages and circulating immune cells. The 3D-SoC model developed in this study represents a powerful research tool to better understand the immune cell trafficking to the skin in homeostasis; inflammatory skin diseases including psoriasis and atopic dermatitis; and autoimmune skin disorders including those pertinent to skin appendages, e.g. alopecia areata. The 3D-SoC can also be potentially adapted to include other missing cell types, such as antigen presenting cells [31] and neutrophils, as well as pathogens [32] to study infectious diseases and wound healing in the context of human cells. However, inclusion of human leukocyte antigen-typed and matched cells, or pluripotent stem cell-derived cells, should be considered to avoid undesired immune reactions especially in applications requiring the use of professional antigen presenting cells (e.g. dendritic cells). Finally, in light of the recent literature describing inter/intra—organ heterogeneities of ECs [13, 33, 34], 3D-SoC may provide the opportunity to explore the molecular and functional distinctions of skin-specific vasculature, which remains understudied compared to other organ-specific vasculatures.

## Data Availability

All data that support the findings of this study are included within the article (and any supplementary files).
